# Predictors of anaemia among adolescent schoolchildren of Ghana

**DOI:** 10.1017/jns.2020.35

**Published:** 2020-09-18

**Authors:** Lucas Gosdin, Katie Tripp, Abraham B. Mahama, Kate Quarshie, Esi Foriwa Amoaful, Lilian Selenje, Deepika Sharma, Maria Elena Jefferds, Andrea J. Sharma, Ralph D. Whitehead, Parminder S. Suchdev, Usha Ramakrishnan, Reynaldo Martorell, O. Yaw Addo

**Affiliations:** 1 Nutrition and Health Sciences, Laney Graduate School, Emory University, Atlanta, GA, USA; 2 Nutrition Branch, Centers for Disease Control and Prevention, 4770 Buford Hwy NE, Atlanta, GA 30341, USA; 3 UNICEF-Ghana, Accra, Ghana; 4 Ghana Health Service of Ministry of Health, Accra, Ghana; 5 UNICEF-Headquarters, New York, NY, USA; 6 U.S. Public Health Service Commissioned Corps, Atlanta, GA, USA; 7 Hubert Department of Global Health, Rollins School of Public Health, Emory University, Atlanta, GA, USA; 8 Emory Global Health Institute, Atlanta, GA, USA

**Keywords:** Adolescent nutrition, Anaemia, Gender differences in anaemia, Determinants of anaemia, Malaria, Geophagy, Pica, 95 % CIs, 95 % confidence intervals, aPR, adjusted prevalence ratios, GIFTS, Girls’ Iron-Folic acid Tablet Supplementation, Hb, haemoglobin concentration, IFA, iron and folic acid, JHS, junior high schools, SHS, senior high schools

## Abstract

Anaemia is a public health problem in Ghana. We sought to identify factors associated with haemoglobin concentration (Hb) and anaemia among school-attending adolescents. We analysed data from 2948 adolescent girls and 609 boys (10–19 years) selected from 115 schools from regions of Ghana as a secondary analysis of baseline surveys conducted at two time-points. We measured Hb, malaria from capillary blood, anthropometry and used a modified food frequency questionnaire to assess diet. Multivariable linear and Poisson regression models were used to identify predictors of Hb and anaemia. The prevalence of anaemia, malaria and geophagy were 24, 25, and 24 %, respectively, among girls and 13, 27 and 6 %, respectively, among boys. Girls engaging in geophagy had a 53 % higher adjusted prevalence of anaemia and 0⋅39 g/dl lower Hb. There were similar results among those who tested positive for malaria (+52 % anaemia; −0⋅42 g/dl Hb). Among girls, lower anaemia prevalence and higher Hb were associated with consumption of foods rich in haeme iron (−22 %; +0⋅18 g/dl), consumption of iron-fortified cereal/beverages consumed with citrus (−50 %; +0⋅37 g/dl) and being overweight (−22 %; +0⋅22 g/dl). Age was positively associated with anaemia among girls, but negatively associated among boys. Boys who tested positive for malaria had 0⋅31 g/dl lower Hb. Boys who were overweight or had obesity and consumed flour products were also more likely to be anaemic (119 and 56 %, respectively). Factors associated with Hb and anaemia may inform anaemia reduction interventions among school-going adolescents and suggest the need to tailor them uniquely for boys and girls.

## Introduction

Adolescence is a period of physiological and psychosocial change during which nutritional needs are changing and new roles and responsibilities are established^(1,2)^. Puberty increases nutritional requirements due to accelerated growth and sexual maturation^(3)^. This period of increasing independence and maturity presents an excellent opportunity for intervention to improve nutritional status and establish lasting, positive dietary and health practices^(2,4,5)^.

The 2017 Ghana Micronutrient Survey estimated the national prevalence of anaemia to be 26⋅4 % among non-pregnant adolescent girls aged 15–19 years, constituting a problem of moderate public health significance^(6)^. There are no nationally representative estimates of anaemia prevalence among adolescent boys. There are multiple causes of anaemia including micronutrient deficiencies, infection, blood loss, genetic disorders and malaria^(7,8)^. Malaria is linked to anaemia by the destruction of erythrocytes and decreased erythropoiesis caused by the malaria parasite^(9)^. Monotonous, micronutrient-poor diets are commonly consumed in low- and middle-income countries and also contribute to the burden of anaemia^(8)^. In Ghana, the practice of pica (compulsive consumption of non-food substances) has been widely observed among young women mainly in the form of geophagy, eating soil or clay^(10–12)^. While the causal direction of the association has not been established, there is a strong association between pica and anaemia^(12,13)^.

In September 2017, a baseline survey was conducted at the beginning of the school year in Northern and Volta regions as part of an impact evaluation of Phase I of the Girls’ Iron-Folic acid Tablet Supplementation (GIFTS) programme^(14)^. The GIFTS programme includes adolescent health and nutrition education and weekly iron and folic acid (IFA) supplementation targeting adolescent girls (10–19 years) in Ghana. It is primarily carried out through schools but also includes health centre-based implementation. The school-based portion of the programme integrates anaemia-related behaviour change communication into existing school health and nutrition education programmes for all adolescents both boys and girls. Topics include anaemia awareness, diet, IFA supplementation awareness, malaria prevention and control, and menstruation among others. Weekly IFA tablets are distributed to girls by teachers and student leaders. In health centres, adolescent girls not attending school receive anaemia-related behaviour change communication and IFA tablets through community outreach and health centre-based activities. The national scale-up was carried out in three phases, of which the school-based portions of Phases I and III were evaluated. During the programme scale-up, a second baseline was conducted in Upper West, Western and Western North regions in September 2019. Data from the two baseline surveys are used in this study to identify key factors driving anaemia among adolescents, which might be applied to tailor the intervention components for the population. The aetiology of anaemia is complex and varies widely across populations^(15,16)^. Using a cross-sectional study design, we aimed to identify context-specific predictors of haemoglobin concentration (Hb) and anaemia among adolescent girls and boys aged 10–19 years in Ghanaian schools^(17)^.

## Methods

### Sampling

Two Phase I regions (Northern and Volta) were purposively selected by the Ghana Health Service of the Ministry of Health and was based on the existing nutrition and health programming data for these regions. Schools within each of the regions were selected by stratified probability proportional to size sampling^(14)^. Stratified sampling between junior high schools (JHS) and senior high schools (SHS) or equivalent was conducted from each region. Schools were treated as clusters and their size was estimated from the previous year's enrolment. The total primary sampling units (schools) were 837 JHS and 68 SHS in the Northern region and 1225 JHS and 104 SHS in the Volta region. Sixty schools were selected: thirty from each region and fifteen from each stratum (JHS/SHS). Simple random sampling was then followed to select twenty-nine girls from forms 1 and 2 within each school (equivalent to grades 7, 8, 10 and 11 in the North American system). For the Phase I parent evaluation, the sample size was powered to detect a 10 % minimum change in the prevalence of anaemia based on a background prevalence of 40 %^(18)^ with 80 % power and a 95 % significance level with 10 % oversampling to account for non-participation and correcting for an intra-class correlation coefficient as high as 10 % for repeated measures^(19)^. This sample is representative of girls in forms 1 and 2 attending secondary and technical schools in the Northern and Volta regions during the beginning of the 2017 academic year.

Phase III included Upper West, Western, and Western North regions, the last of the regions to be included in the GIFTS programme. Schools from each region were sampled following the same methodology as Phase I. Within these three regions, the total primary sampling units (schools) were 2131 JHS and 180 SHS; fifty-five schools (twenty-nine JHS and twenty-six SHS) were selected. Within each school, thirty-one girls and thirteen boys in forms 1 and 2 were randomly selected. Boys were selected during Phase III due to concerns from stakeholders about their exclusion from anaemia reduction efforts. There was no previous data on the burden of anaemia among adolescent boys. The sample size for the Phase III parent evaluation was calculated the same as in Phase I, except that a background anaemia prevalence of 25 % was used based on newly available information^(6)^. Sampling was not meant to be nationally representative but was intended, by design, to be representative of girls and boys in forms 1 and 2 attending secondary and technical schools in the Upper West, Western and Western North regions during the beginning of the 2019 academic year.

### Data collection

Trained interviewers administered a tablet-based electronic questionnaire to each participant. Survey questions pertaining to this analysis included socio-demographic characteristics, anaemia knowledge and practices, menarche, iron supplementation, geophagy and diet.

A mobile laboratory at each school tested Hb using the HemoCue® 301 (Ängelholm, Sweden) and malaria using the CareStart™ Rapid Diagnostic Test (AccessBio, Somerset, NJ, USA). From the finger of each participant, capillary blood was collected. The first drop was wiped clean. During Phase I, the subsequent —two to three drops of blood were collected onto parafilm. The first portion of the sample was analysed immediately for Hb (within 30 s of the finger stick), and the malaria diagnostic was performed using the remaining blood. During Phase III, 1 ml of capillary blood was collected into a Microtainer® tube containing an anticoagulant, and samples were drawn from the tube onto parafilm for immediate analysis of Hb and malaria infection as described for Phase I. HemoCues® were calibrated using a quality control specimen. Following a week-long training, each phlebotomist was tested and only the strongest candidates were retained for actual data collection. During specimen collection, the timing of collection was recorded to ensure that time for potential oxidation of specimens had been minimised.

Standardised anthropometric protocols were used to measure height with a stadiometer (Shorrboard®, Olney, MD, USA)^(20)^ and weight with a lightweight electronic digital scale (SECA, Hamburg, Germany).

This study was conducted according to the guidelines laid down in the Declaration of Helsinki, and all procedures involving human subjects were approved by the Ghana Health Service ethical review board in the Ministry of Health. Written informed consent was obtained from all parents and verbal assent from students. CDC determined its role was public health practice. A de-identified dataset was used for this secondary analysis.

### Variable definitions

The main outcomes of this analysis were Hb and anaemia. Anaemia was defined using age- and gender-specific Hb cut-off values (children 10–11 years: Hb < 11⋅5 g/dl; girls ≥ 12 years and boys 12–14 years: Hb < 12⋅0 g/dl; boys ≥ 15 years: Hb < 13⋅0 g/dl)^(21)^. Hb was not adjusted for altitude or smoking status. Body mass index (BMI)-for-age *z*-scores (BAZ) were calculated based on the International Obesity Taskforce reference parameters, and cut-offs for thinness, overweight and obesity were applied (thinness: BAZ < −2, overweight: BAZ > +1 and obesity: BAZ > +2)^(22,23)^. Normal weight and thin categories were subsequently combined for all students due to the prevalence of thinness <1 %, and for boys, overweight and obesity were combined due to the prevalence of obesity <1 %. Wealth tertiles were calculated following principal component analysis of household possessions^(24)^. Age in years, reached menarche (yes/no), consumption of an iron-containing supplement (iron, IFA or multiple micronutrient supplement) in the previous 7 d (yes/no) and ‘sometimes’ eating soil or clay (geophagy; yes/no) were self-reported. We divided respondents into three categories based on their anaemia knowledge: (1) never heard of anaemia, (2) only heard of anaemia but no knowledge of causes, prevention or symptoms and (3) heard of anaemia, and knowledge of any causes, prevention or symptoms.

Dietary intake was assessed using a modified food frequency questionnaire (FFQ) with a 24-h recall period. The FFQ was developed in coordination with local nutritionists, pretested and modified for clarity before administration; however, it was not formally validated. Grouping of foods was done based on iron content and bioavailability^(25)^. Rich sources of haeme iron included red meats and organ meats. Fair sources of haeme iron included white meats/poultry, eggs and fish. Non-haeme iron sources included dark green leafy vegetables, legumes and seeds. We created a separate category for fortified cereals and beverages, and another category for flour products. Fortified cereals and beverages included commonly sold products with an iron fortificant on the nutrition label. The two final categories considered were tea and citrus fruits.

### Analytical methods

Means, proportions and their 95 % confidence intervals (95 % CIs) were calculated as descriptive statistics. The Rao–Scott chi-square test and ANOVA with Taylor series variance were used to test for differences in characteristics between girls across the five regions and between boys and girls within Upper West, Western, and Western North regions. Rao–Scott chi-square tests were used to assess bivariate differences in anaemia prevalence by malaria status, geophagy and key dietary variables, stratified by gender.

We assessed associations between Hb and adolescent-level factors such as malaria and dietary practices with multivariable linear regression models. Separate models were built for boys and girls. *A priori* selection of model covariates was based on theoretical considerations. These variables were age, BMI, menarche (for girls), iron supplementation, socioeconomic status, diet, malaria and geophagy^(12,16,26,27)^. We also quantified associations at the lower tail of Hb distributions by examining anaemia prevalence. Poisson models with a log link function were used to estimate adjusted prevalence ratios (aPR, 95 % CI) by the predictors of anaemia. Inhibitors and enhancers of non-haeme iron absorption are found in tea and citrus fruits, respectively^(28)^. We therefore examined how same-day tea and citrus consumption could modify the associations between non-haeme and fortified foods with Hb. We also examined the interactions between geophagy with diet, region with all variables and socioeconomic status with diet and geophagy. Only significant interaction terms were retained in the multivariable models.

For all analyses, post-stratification weighting was applied using the school-level enrolment of girls and boys. We used complex survey procedures in all analyses and Taylor series variance estimation was followed. A statistical significance was set at *α* 0⋅05. All analyses were conducted in SAS 9.4 (SAS Institute, Cary, NC, USA).

## Results

In total, 2983 girls participated, with an overall response rate of 95⋅6 %. Thirty-five girls older than 19 years were removed from the current analysis. The analytical sample was 2948 girls, 10–19 years of age. The mean age was 14⋅2 years for JHS girls and 16⋅8 years among SHS girls, and 93⋅8 % had reached menarche. About 64 % of girls had heard of anaemia and had knowledge of its causes, prevention or symptoms; this was highest in the Volta region and lowest in the Northern region. About 5 % of girls reported consuming iron-containing supplements. Nearly one quarter of girls reported engaging in geophagy. Most reported that they had eaten a fair source of haeme iron (68⋅5 %), a source of non-haeme iron (68⋅3 %) and/or a wheat flour product (59⋅6 %) in the previous 24 h. Dietary variables differed significantly across the surveyed regions. The prevalence of malaria and anaemia were nearly equivalent, 24⋅6  and 23⋅5 %, respectively, although their co-occurrence was only 7⋅6 %. The highest prevalence of malaria was in the Northern region (29⋅6 %), while the lowest prevalence was in the Western region (15⋅0 %). The prevalence of anaemia was not significantly different between any two regions; however, Hb differed significantly by the region, being lowest in the Volta and Western regions (Table 1). In total, 615 boys participated in Upper West, Western and Western North regions with a response rate of 95⋅5 %. Six were removed for age older than 19 years. The analytical sample was 609 boys, 11–19 years of age. The mean age was 14⋅2 years for JHS boys and 16⋅9 years for SHS boys. Nearly 62 % had heard of anaemia and had knowledge of its causes, prevention or symptoms. Age, wealth, anaemia knowledge, pre-intervention iron supplementation, diet, BMI and malaria differed significantly in boys among the three regions (Table 2).

**Table 1.** Characteristics of adolescent girls in Ghanaian Schools in Northern, Upper West, Volta, Western and Western North regions

Characteristics	Overall (*n* 2948)	2017		2019			*P*-value
		Girls					
		Northern (*n* 754)	Volta (*n* 767)	Upper West (*n* 720)	Western (*n* 343)	Western North (*n* 364)	
*Demographics*							
Mean age, years	16⋅4 (16⋅2, 16⋅6)	16⋅8 (16⋅5, 17⋅1)	16⋅3 (16⋅0, 16⋅6)	16⋅4 (15⋅9, 16⋅9)	15⋅6 (15⋅2, 16⋅0)	16⋅5 (16⋅0, 17⋅1)	<0⋅01
Reached menarche, %	93⋅8 (91⋅3, 96⋅3)	94⋅4 (91⋅2, 97⋅5)	95⋅1 (91⋅3, 98⋅9)	91⋅3 (85⋅1, 97⋅6)	92⋅4 (86⋅0, 98⋅8)	95⋅8 (91⋅2, 100⋅0)	0⋅64
Wealth tertile, %							<0⋅01
High	43⋅3 (36⋅4, 50⋅2)	33⋅4 (24⋅5, 42⋅3)	50⋅8 (40⋅5, 61⋅0)	29⋅0 (19⋅0, 39⋅0)	71⋅5 (63⋅3, 79⋅8)	49⋅0 (39⋅8, 58⋅1)	
Middle	32⋅3 (29⋅1, 35⋅5)	32⋅7 (26⋅3, 39⋅0)	33⋅0 (28⋅6, 37⋅4)	35⋅4 (29⋅3, 41⋅5)	23⋅3 (18⋅0, 28⋅6)	35⋅6 (29⋅2, 42⋅1)	
Low	24⋅4 (19⋅4, 29⋅4)	33⋅9 (25⋅2, 42⋅6)	16⋅2 (9⋅8, 22⋅7)	35⋅6 (25⋅7, 45⋅5)	5⋅1 (1⋅2, 9⋅1)	15⋅4 (11⋅1, 19⋅8)	
*Knowledge and practices*							
Anaemia knowledge							<⋅01
Heard of anaemia and knowledge of causes, prevention or symptoms, %	63⋅5 (56⋅7, 70⋅4)	46⋅6 (36⋅3,56⋅8)	84⋅2 (80⋅2,88⋅3)	69⋅2 (59⋅8,78⋅6)	65⋅9 (46⋅2,85⋅7)	59⋅1 (45,73⋅2)	
Heard of anaemia, no knowledge of causes, prevention or symptoms, %	18⋅7 (15⋅2, 22⋅3)	25⋅7 (20⋅5,30⋅9)	7⋅6 (4⋅2,10⋅9)	18⋅3 (11⋅8,24⋅8)	20⋅3 (9⋅2,31⋅3)	19⋅4 (11⋅2,27⋅6)	
Never heard of anaemia, %	17⋅7 (13⋅6, 21⋅9)	27⋅7 (19⋅9,35⋅6)	8⋅2 (4⋅8,11⋅6)	12⋅5 (8,16⋅9)	13⋅8 (4⋅6,23)	21⋅5 (15⋅2,27⋅8)	
Pre-intervention iron supplementation in last 7 d, %	5⋅2 (3⋅5, 6⋅9)	4⋅9 (1⋅0, 8⋅7)	3⋅8 (1⋅7, 5⋅9)	5⋅3 (1⋅9, 8⋅8)	8⋅0 (3⋅4, 12⋅6)	5⋅6 (3⋅3, 7⋅8)	0⋅56
Sometimes engage in geophagy (yes/no), %	23⋅7 (20⋅5, 26⋅8)	24⋅6 (17⋅8, 31⋅4)	30⋅0 (22⋅6, 37⋅4)	23⋅1 (17⋅3, 28⋅9)	18⋅7 (11⋅0, 26⋅3)	15⋅1 (8⋅4, 21⋅8)	0⋅08
*Diet, consumed in previous day*							
Rich source of haeme iron, %^a^	19⋅2 (15⋅6, 22⋅8)	20⋅6 (14⋅1, 27⋅1)	12⋅4 (8⋅7, 16⋅1)	21⋅9 (9⋅1, 34⋅6)	22⋅7 (17⋅7, 27⋅8)	20⋅0 (16⋅6, 23⋅4)	0⋅21
Fair source of haeme iron, %^b^	68⋅5 (62⋅0, 75⋅1)	56⋅4 (44⋅3, 68⋅5)	93⋅5 (91⋅2, 95⋅7)	34⋅8 (26⋅8, 42⋅8)	91⋅1 (89⋅1, 93⋅1)	87⋅3 (78⋅1, 96⋅5)	<0⋅01
Source of non-haeme iron, %^c^	68⋅3 (63⋅8, 72⋅7)	63⋅6 (55⋅6, 71⋅6)	71⋅5 (66⋅3, 76⋅6)	78⋅4 (71⋅4, 85⋅3)	58⋅1 (48⋅5, 67⋅6)	70⋅7 (60⋅1, 81⋅3)	<0⋅01
Iron-fortified cereal and beverages, %^d^	44⋅1 (37⋅2, 50⋅9)	36⋅0 (23⋅2, 48⋅7)	49⋅9 (38⋅4, 61⋅4)	47⋅7 (39⋅0, 56⋅4)	43⋅8 (22⋅3, 65⋅3)	50⋅9 (42⋅2, 59⋅6)	0⋅32
Wheat flour products, %^e^	59⋅6 (54⋅4, 64⋅8)	52⋅0 (42⋅8, 61⋅2)	74⋅2 (65⋅0, 83⋅4)	53⋅8 (44⋅2, 63⋅5)	55⋅1 (42⋅2, 68⋅0)	69⋅0 (62⋅4, 75⋅6)	<0⋅01
Tea, %	13⋅7 (10⋅2, 17⋅1)	21⋅4 (12⋅5, 30⋅3)	8⋅6 (0⋅3, 16⋅9)	17⋅5 (12⋅1, 22⋅8)	3⋅4 (0⋅5, 6⋅4)	6⋅5 (1⋅4, 11⋅5)	<0⋅01
Citrus fruit, %^f^	23⋅6 (19⋅7, 27⋅5)	19⋅4 (13⋅4, 25⋅5)	30⋅0 (23⋅7, 36⋅2)	12⋅5 (6⋅2, 18⋅8)	21⋅2 (16⋅1, 26⋅2)	47⋅2 (35⋅5, 59⋅0)	<0⋅01
*Body composition*							
Thin, %^g^	0⋅8 (0⋅3, 1⋅3)	0⋅6 (0⋅0, 1⋅2)	1⋅5 (0⋅4, 2⋅6)	0⋅3 (0⋅0, 0⋅7)	1⋅3 (0⋅0, 3⋅3)	0⋅2 (0⋅0, 0⋅4)	0⋅11
Normal weight, %^h^	77⋅2 (74⋅8, 79⋅6)	79⋅0 (73⋅7, 84⋅3)	74⋅3 (67⋅9, 80⋅8)	74⋅1 (70⋅5, 77⋅7)	78⋅5 (75⋅6, 81⋅3)	81⋅8 (78⋅6, 85⋅0)	0⋅15
Overweight, %^i^	18⋅9 (16⋅7, 21⋅0)	18⋅6 (13⋅7, 23⋅4)	19⋅4 (13⋅8, 25⋅0)	22⋅9 (19⋅6, 26⋅1)	15⋅5 (10⋅4, 20⋅6)	15⋅5 (12⋅7, 18⋅4)	0⋅22
Obesity, %^j^	3⋅0 (2⋅1, 4⋅0)	1⋅9 (0⋅6, 3⋅2)	4⋅7 (2⋅9, 6⋅4)	2⋅3 (1⋅0, 3⋅5)	4⋅8 (2⋅0, 7⋅5)	2⋅4 (0⋅5, 4⋅4)	0⋅02
*Biological measures*							
Malaria, %	24⋅6 (21⋅4, 27⋅9)	29⋅6 (24⋅9, 34⋅4)	20⋅9 (12⋅5, 29⋅2)	27⋅6 (22⋅3, 32⋅9)	15⋅0 (9⋅7, 20⋅4)	24⋅0 (19⋅1, 28⋅9)	<0⋅01
Anaemia, %^k^	23⋅5 (20⋅0, 27⋅0)	24⋅5 (16⋅0, 33⋅0)	26⋅0 (19⋅5, 32⋅4)	21⋅2 (17⋅7, 24⋅7)	26⋅6 (21⋅1, 32⋅0)	15⋅6 (12⋅0, 19⋅2)	0⋅19
Co-occurring anaemia and malaria	7⋅6 (5⋅8, 9⋅4)	9⋅4 (5⋅7, 13⋅1)	5⋅7 (2⋅6, 8⋅7)	10⋅0 (7⋅3, 12⋅8)	6⋅1 (1⋅0, 11⋅2)	3⋅4 (1⋅2, 5⋅7)	0⋅08
Mean haemoglobin concentration, g/dl	12⋅7 (12⋅6, 12⋅8)	12⋅9 (12⋅6, 13⋅2)	12⋅5 (12⋅4, 12⋅7)	12⋅7 (12⋅5, 12⋅8)	12⋅5 (12⋅3, 12⋅7)	12⋅9 (12⋅8, 13⋅0)	<0⋅01

*Note*: Values are weighted. Complex survey procedures used to account for clustering.

a Red meats and organ meats.

b White meats/poultry, eggs and fish.

c Dark green leafy vegetables, legumes and seeds.

d Fortified cereals such as Nestle Cerelac and beverages such as Nido and Milo.

e Breads, pies and cakes made with wheat flour.

f Oranges, lemons and sour sap.

g BMI for age (BAZ) < −2 sd from mean of International Obesity Taskforce reference.

h BAZ between −2 sd and +1 sd.

i BAZ between >+1 sd and +2 sd.

j BAZ >+2 sd.

k Anaemia defined using age-/sex-specific haemoglobin concentration cut-off values (children 10–11 years: Hb < 11⋅5 g/dl; girls ≥ 12 years and boys 12–14 years: Hb <12⋅0 g/dl; boys ≥ 15 years: Hb < 13⋅0 g/dl). There were fourteen cases of severe anaemia among girls (Hb < 8⋅0 g/dl).

**Table 2.** Characteristics of adolescent boys in Ghanaian Schools in Upper West, Western and Western North regions

Characteristic	Overall (*n* 614)	2019			*P*-value
		Boys			
		Upper West (*n* 315)	Western (*n* 138)	Western North (*n* 161)	
*Demographics*					
Mean age, years	16⋅5 (16⋅1, 16⋅9)	16⋅9 (16⋅5, 17⋅4)	15⋅7 (15⋅1, 16⋅2)	16⋅6 (16⋅0, 17⋅2)	<0⋅01
Wealth tertile, %					<0⋅01
High	47⋅3 (32⋅3, 62⋅2)	27⋅6 (20⋅3, 35⋅0)	85⋅5 (72⋅4, 98⋅5)	46⋅4 (34⋅8, 58⋅0)	
Middle	28⋅3 (19⋅7, 37⋅0)	34⋅8 (26⋅7, 42⋅9)	9⋅7 (0⋅9, 18⋅5)	35⋅9 (28⋅6, 43⋅1)	
Low	24⋅4 (15⋅9, 32⋅9)	37⋅6 (29⋅3, 45⋅9)	4⋅8 (0⋅0, 9⋅8)	17⋅7 (9⋅4, 26⋅0)	
*Knowledge and practices*					
Anaemia knowledge					<0⋅01
Heard of anaemia and knowledge of causes, prevention or symptoms, %	61⋅9 (53⋅9, 69⋅9)	72⋅8 (64⋅4, 81⋅3)	50⋅7 (43⋅2, 58⋅2)	50⋅3 (38⋅6, 62⋅1)	
Heard of anaemia, no knowledge of causes, prevention or symptoms, %	21⋅2 (15⋅7, 26⋅7)	14⋅5 (9⋅7, 19⋅4)	28⋅7 (19⋅9, 37⋅5)	27⋅6 (16⋅1, 39⋅0)	
Never heard of anaemia, %	16⋅9 (12⋅5, 21⋅2)	12⋅6 (6⋅8, 18⋅5)	20⋅7 (15⋅8, 25⋅5)	22⋅1 (14⋅9, 29⋅3)	
Pre-intervention iron supplementation in last 7 d, %	4⋅4 (1⋅5, 7⋅4)	2⋅5 (0⋅3, 4⋅7)	11⋅6 (8⋅3, 14⋅9)	0⋅1 (0⋅0, 0⋅4)	<0⋅01
Sometimes engage in geophagy (yes/no), %	6⋅3 (3⋅5, 9⋅0)	7⋅0 (3⋅7, 10⋅2)	3⋅4 (0⋅0, 7⋅8)	8⋅1 (2⋅8, 13⋅4)	0⋅33
*Diet, consumed in previous day*					
Rich source of haeme iron, %^a^	17⋅4 (11⋅5, 23⋅3)	15⋅8 (6⋅0, 25⋅5)	18⋅9 (10⋅8, 26⋅9)	19⋅5 (10⋅8, 28⋅2)	0⋅79
Fair source of haeme iron, %^b^	57⋅1 (46⋅0, 68⋅1)	34⋅1 (26⋅6, 41⋅6)	83⋅5 (74⋅8, 92⋅2)	78⋅2 (60⋅9, 95⋅5)	<0⋅01
Source of non-haeme iron, %^c^	73⋅6 (66⋅7, 80⋅6)	80⋅4 (74⋅4, 86⋅5)	59⋅7 (50⋅2, 69⋅2)	74⋅8 (66⋅1, 86⋅4)	<0⋅01
Iron-fortified cereal and beverages, %^d^	48⋅4 (34⋅8, 62⋅1)	47⋅8 (36⋅2, 59⋅4)	60⋅9 (22⋅9, 98⋅9)	34⋅9 (23⋅6, 46⋅2)	0⋅35
Wheat flour products, %^e^	60⋅7 (51⋅3, 70⋅2)	53⋅8 (40⋅4, 67⋅2)	73⋅8 (61⋅5, 86⋅1)	61⋅1 (50⋅6, 71⋅6)	0⋅02
Tea, %	13⋅4 (8⋅2, 18⋅7)	19⋅8 (12⋅3, 27⋅2)	6⋅7 (0⋅2, 13⋅2)	6⋅9 (1⋅4, 12⋅4)	<0⋅01
Citrus fruit, %^f^	17⋅6 (10⋅3, 24⋅9)	8⋅4 (3⋅1, 13⋅7)	12⋅6 (0⋅4, 24⋅8)	45⋅0 (33⋅1, 56⋅9)	<0⋅01
*Body composition*					
Thin, %^g^	1⋅4 (0⋅3, 2⋅5)	0⋅2 (0⋅0, 0⋅5)	0	5⋅8 (3⋅2, 8⋅3)	–
Normal weight, %^h^	90⋅2 (85⋅3, 95⋅1)	93⋅3 (87⋅0, 99⋅5)	84⋅9 (75⋅4, 94⋅4)	89⋅6 (86⋅1, 93⋅1)	0⋅11
Overweight/obesity^i^	6⋅9 (2⋅2, 11⋅6)	4⋅1 (1⋅0, 7⋅2)	15⋅0 (5⋅5, 24⋅5)	3⋅6 (0⋅6, 6⋅6)	<0⋅01
*Biological measures*					
Malaria, %	26⋅8 (18⋅6, 34⋅9)	35⋅3 (27⋅8, 42⋅8)	11⋅1 (0⋅8, 21⋅5)	26⋅6 (19⋅1, 34⋅1)	<0⋅01
Anaemia, %^j^	13⋅1 (8⋅9, 17⋅3)	10⋅9 (6⋅7, 15⋅0)	16⋅7 (5⋅1, 28⋅4)	13⋅8 (9⋅9, 17⋅8)	0⋅38
Co-occurring anaemia and malaria	4⋅2 (2⋅0, 6⋅4)	5⋅7 (2⋅5, 8⋅9)	2⋅4 (0⋅0, 5⋅9)	3⋅0 (0⋅0, 6⋅1)	0⋅29
Mean haemoglobin concentration, g/dl	14⋅2 (14⋅0, 14⋅4)	14⋅4 (14⋅2, 14⋅7)	13⋅9 (13⋅5, 14⋅2)	14⋅2 (13⋅8, 14⋅6)	0⋅06

*Note*: Values are weighted. Complex survey procedures used to account for clustering.

a Red meats and organ meats.

b White meats/poultry, eggs and fish.

c Dark green leafy vegetables, legumes and seeds.

d Fortified cereals such as Nestle Cerelac and beverages such as Nido and Milo.

e Breads, pies and cakes made with wheat flour.

f Oranges, lemons and sour sap.

g BMI for age (BAZ) <−2 sd from mean of International Obesity Taskforce reference.

h BAZ between −2 sd and +1 sd.

i BAZ >+1 sd.

j Anaemia defined using age-/sex-specific haemoglobin concentration cut-off values (children 10–11 years: Hb < 11⋅5 g/dl; girls ≥ 12 years and boys 12–14 years: Hb < 12⋅0 g/dl; boys ≥ 15 years: Hb < 13⋅0 g/dl). There were fourteen cases of severe anaemia among girls (Hb < 8⋅0 g/dl).

**Fig. 1.**
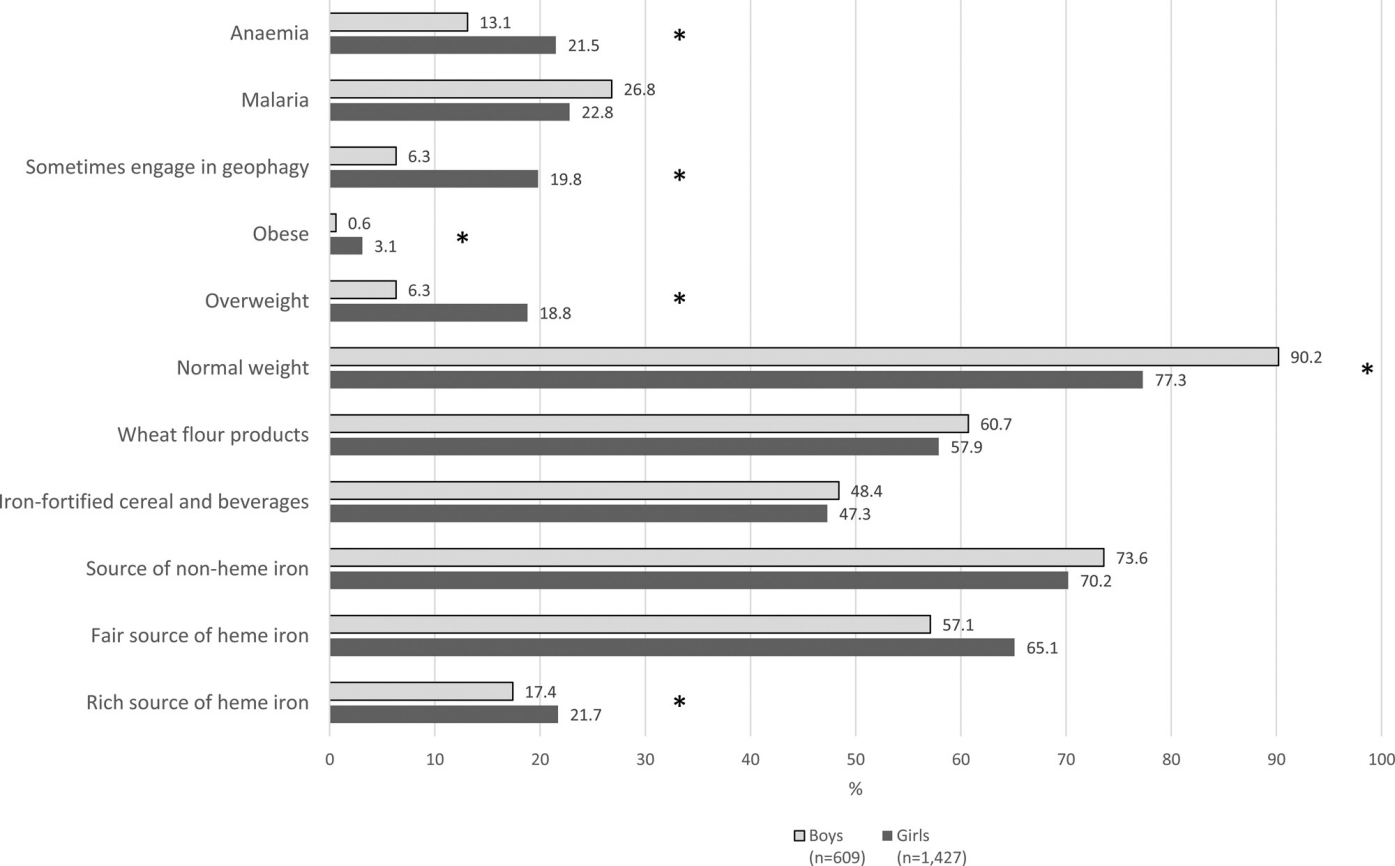
Characteristics of adolescent girls and boys in Ghanaian Schools in Upper West, Western and Western North regions. *Note*: *P < ⋅05. Values are weighted. Complex survey procedures used to account for clustering. Data for boys only available for Upper West, Western and Western North regions. Rich sources of haeme iron included red meats and organ meats; fair sources of haeme iron included white meats/poultry, eggs and fish; sources of non-haeme iron included dark green leafy vegetables, legumes and seeds; fortified foods included cereals such as Nestle Cerelac and beverages such as Nido and Milo; anaemia was defined using age-/sex-specific haemoglobin concentration cut-off values (children 10–11 years: Hb < 11⋅5 g/dl; girls ≥ 12 years and boys 12–14 years: Hb < 12⋅0 g/dl; boys ≥ 15 years: Hb < 13⋅0 g/dl).

**Fig. 2.**
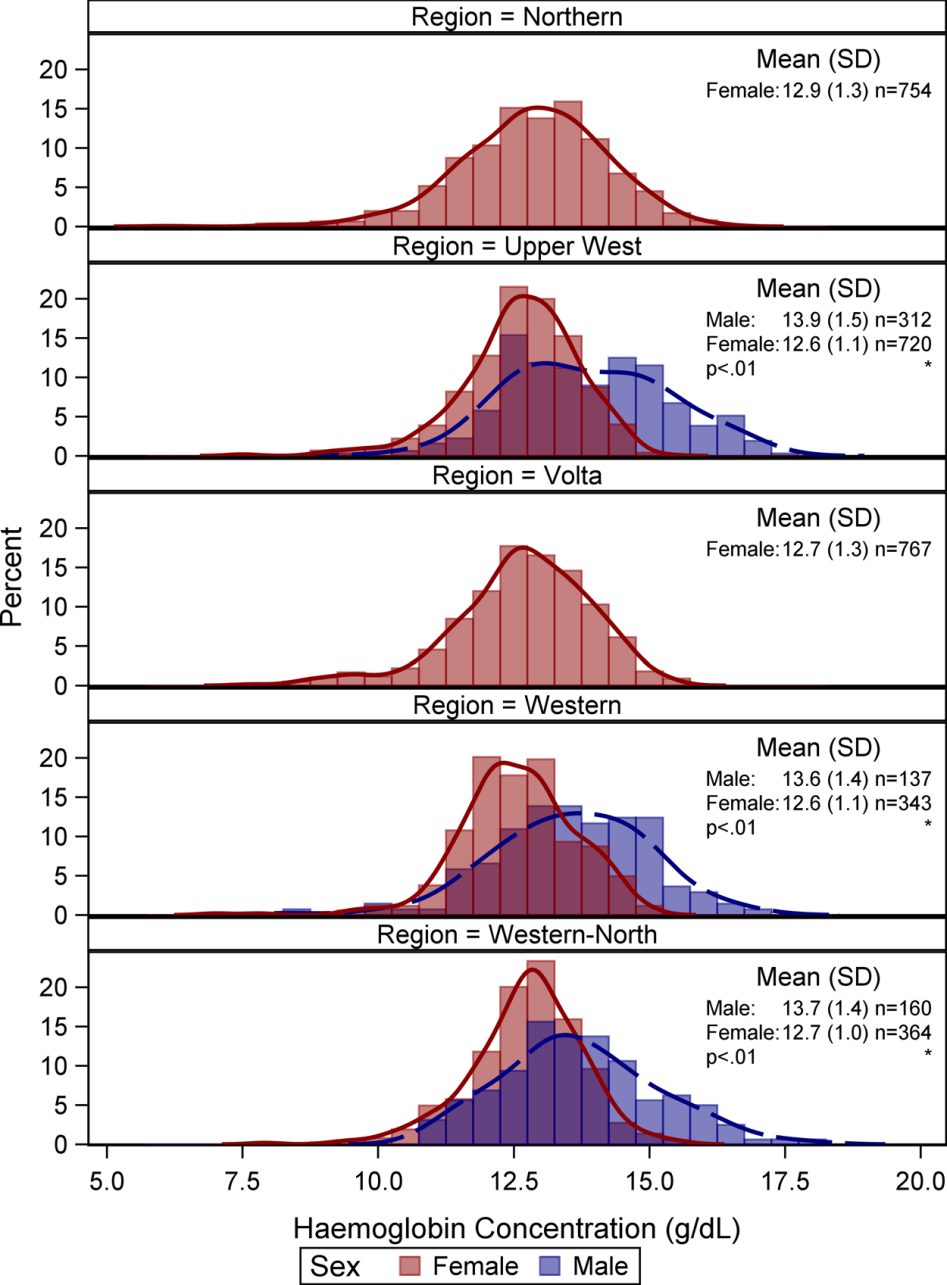
Distribution of haemoglobin concentration among adolescent girls and boys in Ghanaian Schools by regions (*n* 2948). *Note*: Unweighted descriptive statistics and histograms. *P < 0⋅01. Complex survey procedures and weights used to account for clustering in testing the differences in mean haemoglobin concentration between girls and boys.

Compared with the 1427 girls assessed in the same three regions, a lower proportion of boys (13⋅1 %) than girls (21⋅5 %) had anaemia (*P* < 0⋅01), but boys had a similar prevalence of malaria (26⋅8 %) as girls (22⋅8 %) (*P* 0⋅27). More girls engaged in geophagy than boys (19⋅8 *v.* 6⋅3 %, respectively; *P* < 0⋅01). Ninety percent of boys were normal weight, while 77⋅3 % of girls were normal weight (*P* < 0⋅01). The composition of the diets of boys and girls was similar, except that rich sources of haeme iron were consumed by more girls (21⋅7 %) than boys (17⋅4 %) (Fig. 1). Mean Hb was significantly higher among boys than girls overall in these three regions (boys 14⋅2 g/dl *v*. girls 12⋅7 g/dl; *P* < ⋅01) and within each of the regions where both genders were surveyed (Fig. 2).

**Fig. 3.**
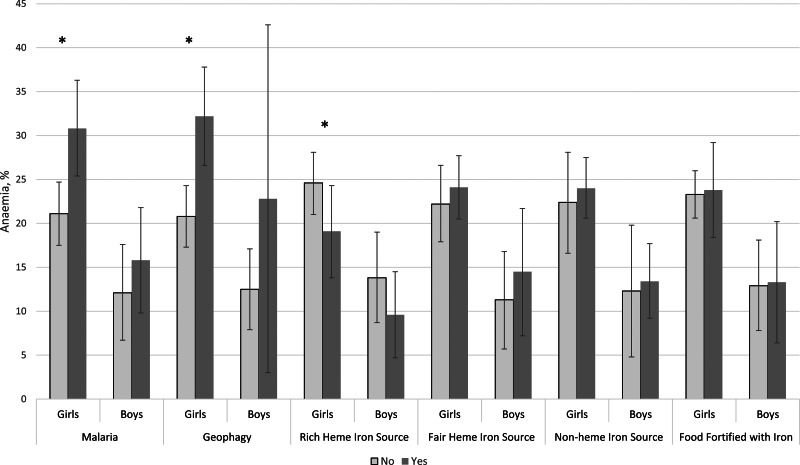
Unadjusted prevalence of anaemia among adolescent girls and boys in Ghanaian Schools by malaria, geophagy and selected dietary variables, Northern, Upper West, Volta, Western and Western North regions (*n* 2948). *Note*: *P < 0⋅05. Values are weighted. Complex survey procedures used to account for clustering. Data for boys only available for Upper West, Western and Western North regions. Rich sources of haeme iron included red meats and organ meats; fair sources of haeme iron included white meats/poultry, eggs and fish; sources of non-haeme iron included dark green leafy vegetables, legumes and seeds; fortified foods included cereals such as Nestle Cerelac and beverages such as Nido and Milo; anaemia was defined using age-/sex-specific haemoglobin concentration cut-off values (children 10–11 years: Hb < 11⋅5 g/dl; girls ≥ 12 years and boys 12–14 years: Hb < 12⋅0 g/dl; boys ≥ 15 years: Hb < 13⋅0 g/dl).

Fig. 3 shows the unadjusted prevalence of anaemia according to malaria status, geophagy and indicators of dietary iron intake. The prevalence of anaemia was 9⋅7 percentage points (*P* < 0⋅01) higher among girls with a positive malaria test (30⋅8 *v*.21⋅1 %). Similarly, anaemia was 11⋅4 percentage points (*P* < 0⋅01) higher among girls who engaged in geophagy (32⋅2 *v*. 20⋅8 %). The prevalence of anaemia was 5⋅5 percentage points (*P* 0⋅02) lower among girls who consumed a rich source of haeme iron (19⋅1 *v.* 24⋅6 %). The prevalence of anaemia among girls did not differ by any other dietary iron intake factors, nor did it vary by other covariates including menarche, BMI and reported consumption of iron-containing supplements in the past week (not shown). The prevalence of anaemia also varied by wealth among girls: highest wealth tertile 26⋅6 %, middle 20⋅1 % and lowest 22⋅9 % (*P* 0⋅04; data not shown). None of these variables was associated with the prevalence of anaemia among boys in unadjusted analyses.

Results of a multivariable model examining predictors of Hb are shown in Table 3. Geophagy was inversely associated with Hb such that girls who reported engaging in the practice had on average 0⋅39 g/dl lower Hb relative to their peers who do not practice geophagy. Similarly, positive malaria infection status was negatively associated with Hb (*β* −0⋅42 g/dl). Reported consumption of a rich source of haeme iron was associated with higher Hb (*β* +0⋅18 g/dl) as was reported consumption of an iron-fortified food or beverage with a citrus fruit within 24 h (*β* +0⋅37 g/dl); there was a significant interaction between the two foods. Girls who were overweight, relative to those with normal weight or thinness, had a higher Hb (*β* +0⋅22 g/dl). Positive malaria infection status was negatively associated with Hb among boys (*β* −0⋅31 g/dl). Hb increased 0⋅35 g/dl for each additional year of age in boys.

**Table 3.** Multivariable linear regression model of the predictors of haemoglobin concentration among adolescent girls and boys in Ghanaian Schools in Northern, Upper West, Volta, Western and Western North regions

Characteristic	Girls (*n* 2860)		Boys (*n* 609)^§^	
	Estimate, g/dl (95 % CI)^a^	*P*-value	Estimate, g/dl (95 % CI)^a^	*P*-value
Geophagy	−0⋅39 (−0⋅60, −0⋅18)	<0⋅01	−0⋅34 (−1⋅10, 0⋅42)	0⋅37
Malaria	−0⋅42 (−0⋅56, −0⋅27)	<0⋅01	−0⋅31 (−0⋅60, −0⋅02)	0⋅04
Rich source of haeme iron^b^	0⋅18 (0⋅00, 0⋅36)	0⋅05	0⋅29 (−0⋅31, 0⋅90)	0⋅34
Fair source of haeme iron^c^	−0⋅11 (−0⋅25, 0⋅04)	0⋅15	−0⋅01 (−0⋅30, 0⋅27)	0⋅92
Source of non-haeme iron^d^	0⋅00 (−0⋅17, 0⋅17)	0⋅98	−0⋅08 (−0⋅40, 0⋅25)	0⋅63
Iron-fortified cereal and beverages^e^	–	–	−0⋅12 (−0⋅39, 0⋅15)	0⋅38
Consumed with citrus^f^	0⋅37 (0⋅09, 0⋅65)	0⋅01	–	–
Consumed without citrus^f^	−0⋅08 (−0⋅23, 0⋅07)	0⋅3	–	–
Flour products^g^	−0⋅04 (−0⋅17, 0⋅08)	0⋅48	−0⋅17 (−0⋅46, 0⋅12)	0⋅24
Normal weight/thin^h^	Ref.	–	Ref.	–
Overweight^i^	0⋅22 (0⋅03, 0⋅42)	0⋅03	−0⋅05 (−0⋅51, 0⋅41)	0⋅83
Obesity^j^	0⋅10 (−0⋅16, 0⋅36)	0⋅45	–	–
Pre-intervention iron supplementation in last 7 d	−0⋅19 (−0⋅58, 0⋅21)	0⋅21	0⋅80 (−0⋅06, 1⋅66)	0⋅07
Age, years	−0⋅02 (−0⋅08, 0⋅03)	0⋅40	0⋅35 (0⋅27, 0⋅44)	<0⋅01
Menarche	−0⋅05 (−0⋅41, 0⋅31)	0⋅77	–	–
Wealth				
High	−0⋅18 (−0⋅40, 0⋅04)	0⋅11	−0⋅11 (−0⋅40, 0⋅17)	0⋅42
Middle	0⋅07 (−0⋅12, 0⋅27)	0⋅45	−0⋅01 (−0⋅33, 0⋅31)	0⋅94
Low	Ref.	–	Ref.	–

*Note*: Values are weighted. Complex survey procedures used to account for clustering.

a Haemoglobin concentration (g/dl).

b Red meats and organ meats.

c White meats/poultry, eggs and fish.

d Dark green leafy vegetables, legumes and seeds.

e Fortified cereals such as Nestle Cerelac and beverages such as Nido and Milo.

f Oranges, lemons, and sour sap. Interaction with iron-fortified foods.

g Breads, pies and cakes made with wheat flour.

h BMI for age (BAZ) <+1 sd from mean of International Obesity Taskforce reference.

i BAZ between >+1 sd and +2 sd.

j BAZ >+2 sd. Overweight and obesity were merged for boys due to a very low proportion with obesity (<1 %).

§ Boys data only available in Upper West, Western, and Western North regions.

Results of a multivariable model examining predictors of anaemia are shown in Table 4. Geophagy was positively associated with anaemia such that the prevalence of anaemia was 53 % higher (aPR 1⋅53) among girls who engaged in the practice. Positive malaria infection status was also associated with anaemia (aPR 1⋅52). Reported consumption of a rich source of haeme iron and an iron-fortified food or beverage with a citrus fruit were, respectively, associated with a 22 % (aPR 0⋅78) and 50 % (aPR 0⋅50) lower prevalence of anaemia. Girls who were overweight had a 22 % (aPR 0⋅78) lower prevalence of anaemia relative to girls with normal weight or thinness. There was an average 9 % (aPR 1⋅09) higher prevalence of anaemia for each addition year of age, and girls from the highest wealth tertile had a 33 % (aPR 1⋅33) higher prevalence of anaemia relative to girls from the lowest wealth tertile. Among boys, only age, BMI and consumption of wheat flour products were associated with anaemia, controlling for covariates. Boys who consumed flour products had a 56 % (aPR 1⋅56) higher prevalence of anaemia. Boys who were overweight or had obesity relative to normal weight or thin had a 119 % (aPR 2⋅19) higher prevalence of anaemia. There was an average 16 % (aPR 0⋅84) lower prevalence of anaemia for each year older a group of boys was.

**Table 4.** Multivariable Poisson regression model of the predictors of anaemia among adolescent girls and boys in Ghanaian Schools in Northern, Upper West, Volta, Western and Western North regions

	Girls (*n* 2860)		Boys (*n* 609)^§^	
	Prevalence ratio (95 % CI)^a^	*P*-value	Prevalence ratio (95 % CI)^a^	*P*-value
Geophagy	1⋅53 (1⋅26, 1⋅85)	<0⋅01	1⋅62 (0⋅51, 5⋅18)	0⋅42
Malaria	1⋅52 (1⋅27, 1⋅82)	<0⋅01	1⋅39 (0⋅87, 2⋅21)	0⋅17
Rich source of haeme iron^b^	0⋅78 (0⋅63, 0⋅97)	0⋅02	0⋅66 (0⋅30, 1⋅45)	0⋅30
Fair source of haeme iron^c^	1⋅15 (0⋅97, 1⋅36)	0⋅10	1⋅00 (0⋅49, 2⋅04)	0⋅99
Source of non-haeme iron^d^	1⋅13 (0⋅90, 1⋅42)	0⋅29	1⋅18 (0⋅67, 2⋅07)	0⋅57
Iron-fortified cereal and beverages^e^	–	–	1⋅06 (0⋅59, 1⋅93)	0⋅84
Consumed with citrus^f^	0⋅50 (0⋅31, 0⋅81)	<0⋅01	–	–
Consumed without citrus^f^	1⋅13 (0⋅95, 1⋅33)	0⋅17	–	–
Flour products^g^	0⋅9 (0⋅79, 1⋅03)	0⋅11	1⋅56 (1⋅00, 2⋅42)	0⋅05
Normal weight/thin^h^	Ref.	–	Ref.	–
Overweight^i^	0⋅78 (0⋅62, 0⋅98)	0⋅03	2⋅19 (1⋅03, 4⋅66)	0⋅04
Obesity^j^	1⋅03 (0⋅68, 1⋅55)	0⋅91	–	–
Pre-intervention iron supplementation in last 7 d	1⋅13 (0⋅75, 1⋅68)	0⋅56	0⋅56 (0⋅06, 4⋅99)	0⋅61
Age, years	1⋅09 (1⋅01, 1⋅16)	0⋅02	0⋅84 (0⋅72, 0⋅99)	0⋅03
Menarche	0⋅88 (0⋅59, 1⋅32)	0⋅54	–	–
Wealth				
High	1⋅33 (1⋅04, 1⋅70)	0⋅03	0⋅87 (0⋅48, 1⋅59)	0⋅66
Middle	0⋅93 (0⋅73, 1⋅19)	0⋅56	0⋅83 (0⋅43, 1⋅62)	0⋅59
Low	Ref.	–	Ref.	–

*Note*: Values are weighted. Complex survey procedures used to account for clustering.

a Haemoglobin concentration (g/dl).

b Red meats and organ meats.

c White meats/poultry, eggs and fish.

d Dark green leafy vegetables, legumes and seeds.

e Fortified cereals such as Nestle Cerelac and beverages such as Nido and Milo.

f Oranges, lemons and sour sap. Interaction with iron-fortified foods.

g Breads, pies and cakes made with wheat flour.

h BMI for age (BAZ) <+1 sd from mean of International Obesity Taskforce reference.

i BAZ between >+1 sd and +2 sd.

j BAZ >+2 sd. Overweight and obesity were merged for boys due to a very low proportion with obesity (<1 %).

§ Boys data only available in Upper West, Western and Western North regions.

## Discussion

In Northern, Upper West, Volta, Western and Western North regions of Ghana, adolescent schoolgirls have a moderate burden of anaemia, above 20 % but below 40 %^(21)^. The burden of anaemia is somewhat lower among boys but remains a mild public health problem, above 5 % but below 20 %, in the surveyed regions. Previous population-based surveys such as national Demographic and Health Surveys in the region had not included boys, girls in early adolescence or explored predictors of anaemia in the secondary school context. Our findings for girls align with previous analyses of the predictors of anaemia among women of reproductive age in that diet and malaria are associated with anaemia^(29)^, although the emergence of geophagy as a prevalent predictor of anaemia in girls is notable. Since none of the predictors is unique to school-going adolescent girls in these regions, these results may be applicable to other adolescents in Ghana. However, our results underscore the need to examine the context-specific predictors of anaemia, since they differed by gender even within the same setting. This may be due to biological differences and potential underlying health conditions between girls and boys. The significant predictors of mean Hb were also associated with anaemia, suggesting that these predictors act on the entire distribution rather than only those with already low Hb.

Hb was not adjusted for altitude or smoking status as no adjustment is needed for populations living below 1000 m above sea level^(21)^, and smoking is rare among adolescents in Ghana^(30)^. Our results show that nearly 40 % of students had no knowledge of the causes, prevention or symptoms of anaemia. Knowledge of anaemia did not predict Hb or anaemia in this study population nor did its inclusion in adjusted models alters other measures of association. However, the gap in anaemia-related knowledge may indicate gaps in health and nutrition education where there is potential to improve anaemia prevention and control behaviours. Only 5 % of girls had taken an iron-containing supplement in the previous 7 d, another opportunity for intervention given the WHO recommendations for supplementation^(31)^.

We also found that diet was associated with Hb and anaemia among girls. We chose to separate animal source foods into rich and fair sources of haeme iron because of their marked differences in content of highly bioavailable iron^(25)^. Fair sources of haeme iron were more commonly eaten, but rich sources of haeme iron were associated with improved Hb and anaemia status. Because consumption of rich sources of haeme iron is low, increased consumption of foods such as red and organ meat may affect Hb and anaemia, recognising access may be a limitation. A difference in the effect of iron-fortified products by same-day consumption of citrus fruits was also observed. Ascorbic acid, found in citrus fruits, reduces iron from its ferric to ferrous form making it an enhancer of non-haeme iron absorption^(32)^. Given the large proportion of girls and boys who consumed fortified cereals and beverages, promotion of the consumption of citrus may increase the bioavailability of iron in the established diets of Ghanaian schoolchildren.

A national policy is in place mandating wheat flour fortification with Vitamin A, folic acid, Vitamin B12, thiamin, riboflavin, niacin, iron and zinc^(33)^. However, reported wheat flour consumption was not associated with Hb or anaemia among girls. It is not likely that they are consuming too little of the wheat flour products as they are common in Ghana – 60 % of study participants had consumed them in the previous day (no significant difference between boys and girls). Challenges with adequate fortification of wheat flour may be the reason for this lack of association as evaluations have shown that most wheat flour in Ghana is not adequately fortified^(6,33)^. Among boys, however, there was significant positive association between consumption of flour products and anaemia. Indicators of body mass had opposite relationships with anaemia among girls and boys. Among boys, we hypothesise that increased consumption of nutrient-poor calorie-dense foods such as the breads and pies (categorised as flour products) might be associated with overweight/obesity and displaces better sources of iron and other micronutrients in the diets of boys. We are unsure of the reasons for the observed protective effect of overweight on anaemia among girls. Wealth among girls was positively associated with anaemia. As a social determinant of health, wealth is often inversely associated with anaemia; however, in societies undergoing the nutrition transition, wealth may also lead to a shift from traditional foods towards packaged foods that are poor sources of micronutrients^(34)^. Age also had opposite effects on anaemia by gender, likely due to the differential effects of puberty on blood loss and testosterone production^(35)^.

Geophagy presents another potential opportunity for intervention. Approximately one quarter of schoolgirls engaged in geophagy, and this did not differ by school level, region or socioeconomic status. It did differ significantly by gender. Geophagy, a subset of the practice of pica, is not well understood, and the causal direction of its relationship to anaemia has not been established^(12)^. It is widely observed among pregnant women in Ghana but less-often studied among adolescents, and its relationship with anaemia warrants further study^(10)^.

Malaria infection was associated with Hb and anaemia among girls and with Hb among boys. Ghana is a malaria-endemic country, and the surveys were conducted during September, the peak transmission season^(36)^. This finding underscores that malaria prevention is an essential part of anaemia control in Ghana^(37)^. However, the low co-occurrence of the two conditions indicates that malaria is not the only driver of anaemia in the population.

This study has several strengths. The surveys are representative of girls in both late and early adolescence within five regions, and inclusive of students of both genders within three regions. The relatively large sample of girls improves the precision of estimates, and high participation rates reduce the potential for selection bias. This study advances knowledge within an understudied population that is receiving increasing attention concerning health and nutrition^(3,38)^.

The study limitations include the cross-sectional design, which hinders the ability to ascertain temporal sequence between study outcome and model predictors. Due to time and cost constraints, we used an imprecise measure (relative to a 24 h dietary recall or a weighted food record) of diet that paid special attention to sources of iron. The method used may not reflect usual dietary intake within the individual^(39)^. We are not able to determine the level of parasitaemia load from the malaria rapid diagnostic test nor do we have information about recent treatment, which would have improved the sensitivity of this measure. We also slightly modified the procedure for collecting capillary Hb between the Phase I and Phase III samples, though this is not expected to cause differences in the measures due to equivalent exposure to oxygen^(40)^. The present study is also missing potentially important predictors of anaemia such as micronutrient deficiencies, inflammation, illness, other parasitic infections and diseases, and haemoglobinopathies^(16, 26)^, which would contribute to a fuller picture of the drivers of anaemia in the population. Our results are also limited to a specific season, and predictors of anaemia may differ throughout the year. Finally, the present study did not assess out-of-school adolescents. Further investigation is needed to understand if the predictors of anaemia and haemoglobin differ for these populations.

Approximately one in four adolescent schoolgirls in Ghana had anaemia. Malaria infection, geophagy and consumption of highly bioavailable iron are associated with haemoglobin concentration among adolescent girls in Ghanaian schools. These modifiable factors may be useful targets for strengthening the Ghanaian anaemia reduction strategy for adolescent girls. Other determinants of anaemia including age, wealth and body mass, and their differential effects by gender may be useful for tailoring anaemia reduction efforts to reach both adolescent schoolgirls and schoolboys.
